# Effects of Natural and Synthetic Astaxanthin on Growth, Body Color, and Transcriptome and Metabolome Profiles in the Leopard Coralgrouper (*Plectropomus leopardus*)

**DOI:** 10.3390/ani13071252

**Published:** 2023-04-04

**Authors:** Junpeng Zhang, Changxu Tian, Kecheng Zhu, Yong Liu, Can Zhao, Mouyan Jiang, Chunhua Zhu, Guangli Li

**Affiliations:** 1 Fisheries College, Guangdong Ocean University, Zhanjiang 524088, China; 2 Guangdong Research Center on Reproductive Control and Breeding Technology of Indigenous Valuable Fish Species, Guangdong Provincial Engineering Laboratory for Mariculture Organism Breeding, Guangdong Provincial Key Laboratory of Aquatic Animal Disease Control and Healthy Culture, Zhanjiang 524088, China; 3 Southern Marine Science and Engineering Guangdong Laboratory (Zhanjiang), Zhanjiang 524088, China; 4 Key Laboratory of South China Sea Fishery Resources Exploitation and Utilization, Ministry of Agriculture and Rural Affairs, South China Sea Fisheries Research Institute, Chinese Academy of Fishery Sciences, Guangzhou 510300, China

**Keywords:** *Plectropomus leopardus*, astaxanthin, coloration, transcriptome, metabolome

## Abstract

**Simple Summary:**

The leopard coralgrouper (*Plectropomus leopardus*) is highly valuable in the ornamental fish market owing to its bright red color. Astaxanthin is commonly used as a red colorant in aquaculture. To investigate the genetic basis of red color formation in *P. leopardus*, we compared the effects of natural and synthetic astaxanthin on *P. leopardus* color and transcriptome and metabolome profiles. Natural astaxanthin had a superior effect on color compared to that of synthetic astaxanthin, as evidenced by increases in redness and yellowness. Carotenoid- and melanin-related genes were differentially expressed between groups treated with natural astaxanthin, synthetic astaxanthin, and no astaxanthin. These results provide a theoretical basis for color enhancement during the culture of *P. leopardus*.

**Abstract:**

Natural and synthetic astaxanthin can promote pigmentation in fish. In this study, the effects of dietary astaxanthin on growth and pigmentation were evaluated in leopard coralgrouper (*Plectropomus leopardus*). Fish were assigned to three groups: 0% astaxanthin (C), 0.02% natural astaxanthin (HP), and 0.02% synthetic astaxanthin (AS). Brightness (L*) was not influenced by astaxanthin. However, redness (a*) and yellowness (b*) were significantly higher for fish fed astaxanthin-containing diets than fish fed control diets and were significantly higher in the HP group than in the AS group. In a transcriptome analysis, 466, 33, and 32 differentially expressed genes (DEGs) were identified between C and HP, C and AS, and AS and HP, including various pigmentation-related genes. DEGs were enriched for carotenoid deposition and other pathways related to skin color. A metabolome analysis revealed 377, 249, and 179 differential metabolites (DMs) between C and HP, C and AS, and AS and HP, respectively. In conclusion, natural astaxanthin has a better coloration effect on *P. leopardus*, which is more suitable as a red colorant in aquaculture. These results improve our understanding of the effects of natural and synthetic astaxanthin on red color formation in fish.

## 1. Introduction

Body color is one of the most striking phenotypic characteristics of fish and plays important roles in predation, camouflage, immunity, and courtship [1,2]. The formation of body color is determined by combinations and distributions of different types of pigment cells [3]. Body color can be divided into pigment color and structural color according to the mechanisms underlying color development [4]. The combination of these two pigment patterns ultimately determines the color of fish [5]. Six chromatophores have been identified in teleosts, including melanophores, erythrophores, xanthophores, iridophores, leucophores, and cyanophores [6]. The distribution of different chromatophores leads to color differences among sites on the fish body. For example, melanophore, xanthophore, and iridophore pigment cells have been found in zebrafish [7]. The types of pigment cells differ between the dark and light areas of this species [8].

Fish color patterns are closely related to genetic, physiological, environmental, and dietary factors, of which genetic factors are the most critical determinants [9]. Melanin, particularly the eumelanin, is the most widely distributed pigment in various animals, and a series of genes associated with eumelanin have been identified, such as *microphthalmia-associated transcription factor* (*mitf*), *melanocortin receptor 1* (*mc1r*), *agouti signaling protein* (*asip*), *tyrosinase* (*tyr*), *tyrosinase-related protein 1* (*tyrp1*), and *tyrosinase-related protein 2* (*tyrp2*) [10,11]. Carotenoids often contribute to red and yellow in the skin or muscle tissues of fish. Borel et al. found that both *scavenger receptor class B member 1* (*scarb1*) and *cluster determinant 36* (*cd36*) are involved in carotenoid uptake and transport [12]. In *Cyprinus carpio* with *scarb1* knockout, regional red skin fades into white and the astaxanthin content is decreased [13]. The gene *tetratricopeptide repeat domain protein 39B* (*ttc39b*), associated with lipid metabolism in mammals, is highly expressed in the red skin of cichlid fish [14]. The gene *colony-stimulating factor 1* (*csf1*) is associated with the maturation and development of yellow pigment cells and may be involved in the formation of yellow skin in fish [15,16]. The gene *apolipoprotein D* (*apod*) is associated with carotenoid deposition and is up-regulated in the yellow mutant rainbow trout [17]. The *beta-carotene 15,15′-monooxygenase-1* (*bcmo1*) and *beta-carotene 9’,10’-oxygenase* (*bco2*) genes are involved in carotenoid catabolism in mammals and fishes [18]. Furthermore, *cytochrome P450*, *family 2*, *subfamily J*, *polypeptide 19* (*cyp2j19*) may be involved in regulating the conversion of yellow carotenoids to red ketocarotenoids in red feathers of birds [19,20]. Recently, Huang et al. found that *cytochrome P450*, *family 2*, *subfamily AE*, *polypeptide 2* (*cyp2ae2*) also plays an important role in ketocarotenoid accumulation in *Danio albolineatus* erythrophores [21]. However, few studies of these genes have focused on fish. In particular, genes related to the formation of red body color in fish are not well studied.

The formation of various body colors in fish is dependent on melanin, carotenoids, and pteridine. Carotenoids cannot be synthesized in fish and need to be obtained from dietary sources [22]. Therefore, the addition of carotenoids to feed is an important strategy to promote fish coloration. Carotenoids commonly used in aquaculture include synthetic β-carotene, astaxanthin, canthaxanthin, zeaxanthin, and lutein [23]. Astaxanthin, a keto-carotenoid, promotes coloration [24] and improves antioxidant activity [25] and immunity [26]. Astaxanthin tissue deposition in fish with pink/red coloration in integument is greater than that of other carotenoids [27]. In *Amphiprion ocellaris*, feeding astaxanthin results in a more intense red color than that obtained by feeding lutein, as well as a significant increase in the skin carotenoid content [24]. Synthetic astaxanthin and natural astaxanthin are available on the market. Synthetic astaxanthin is obtained by chemical synthesis using chemical raw materials and is a racemic mixture with an optical isomer ratio of 1:2:1 (3S,3′S:3R,3′S:3R,3′R) [25,28]. Natural astaxanthin is obtained from microalgae, such as *Bracteacoccus aggregatus* BM5/15, yeasts *Phaffia rhodozyma*, or crustaceans, such as shrimp and crabs [29,30,31]. The microalgae *Haematococcus lacustris* (formerly *Haematococcus pluvialis*) was reported to have abundant astaxanthin [32]. Astaxanthin of *H. lacustris* exists mainly in esterified form, with a levorotatory structure (3S,3’S) in the spin isomer. The stability and antioxidant activity of astaxanthin extracted from this species were higher than those of synthetic astaxanthin [25,33]. Therefore, *H. lacustris* is one of the best sources of natural astaxanthin. It is worth noting that the uptake and utilization efficiency of esterified/free astaxanthin by fish may vary according to species. For instance, relatively little tissue deposition of natural astaxanthin has been observed in salmon [34]. In contrast, natural astaxanthin showed better effects on color than those of synthetic astaxanthin in *Cyprinus carpio*, *Carassius auratus,* and *Pseudochromis fridmani*, although both improved fish color [35,36].

The leopard coralgrouper (*Plectropomus leopardus*) is a coral reef fish with a bright red body color, luster, and blue spots. Its skin redness is one of the most important economic traits determining its market value [37,38]. Astaxanthin has been shown to be more effective in coloring *P. leopardus* than other carotenoid sources, such as spirulina [39,40]. However, little is known about the effects of different sources of astaxanthin on coloration in *P. leopardus*. In the present study, we compared the effects of natural astaxanthin and synthetic astaxanthin on the growth and pigmentation of *P. leopardus*. Meanwhile, we used transcriptomic and metabolomic approaches to investigate the candidate genes and metabolites that may be involved in the formation of body color, providing a theoretical basis for the mechanism of coloration in *P. leopardus*.

## 2. Materials and Methods

### 2.1. Ethics Statement

All experimental protocols were approved by the Animal Research and Ethics Committee of Guangdong Ocean University (201903003) in this study. The study does not involve endangered or protected species. During the experiments, the experimental fish were euthanized according to the euthanasia procedure described in AVMA Guidelines for the Euthanasia of Animals: 2020 Edition [41].

### 2.2. Experimental Diets

In the experiment, three diets were designed, which were the basal diet without astaxanthin, the synthetic astaxanthin diet with Carophyll^®^ pink powder (AS group) and a natural astaxanthin diet with *H. lacustris* powder (HP group), where the basal diet without astaxanthin was used as a negative control (C group). The preparation of the experimental diets followed the method of Jiang et al. [36]: microcrystalline cellulose was chosen as a filler to prepare the feeds. An amount of 5% microcrystalline cellulose was added to the basal diet. Additionally, for the treatment of the diets for the synthetic astaxanthin group and the natural astaxanthin group, 0.2% Carophyll^®^ pink powder or 0.667% *H. lacustris* powder were selected instead of equal amounts of microcrystalline cellulose, respectively, thus allowing quantification of the astaxanthin content in the diets.

The composition and approximate composition of the experimental diet is shown in Table 1. The basal diet was not supplemented with astaxanthin, and the experimental diets were supplemented with 0.2% of Carophyll^®^ pink powder (10% astaxanthin) and 0.667% of *Haematococcus lacustris* powder (3% astaxanthin) to ensure that the actual content of natural astaxanthin and synthetic astaxanthin was consistent, both at 0.02%. The protein raw materials were crushed, homogenized, sieved, and then mixed thoroughly with other ingredients to produce feed pellets with a diameter of 4.0 mm using a double screw extruder (TSE65, Beijing Modern Yanggong Machinery Technology Development Co., Ltd., Beijing, China). All diets were stored at −20 °C throughout feeding to avoid pigment oxidation.

### 2.3. Experimental Fish and Sample Collection

The experimental fish (7-month-old adult fish), of unknown sex, were purchased from Guangdong Sanhe Lvyuan Aquaculture Co., Ltd., Zhanjiang, China. Fish were placed in an indoor cement pool (3.5 m × 5 m × 0.6 m) and fed basal feed for 14 days to adapt to the experimental environment. After that, 450 healthy adult fish with uniform size (fish average mass: 132.43 ± 6.69 g) were selected and then randomly separated into three groups, each of which was placed in three cages (2 m × 1 m × 1 m). The fish were cultured in a recirculating aquaculture system and fed twice a day (8 am and 4 pm) at 5% of their body weight for 14 weeks. Flowing water is used to ensure that the water contains sufficient oxygen while avoiding stress from low oxygen levels. The mass of experimental fish was measured every seven weeks. After the feeding trial, all fish were starved for 24 h. Nine fish of each group were randomly selected for sample collection after anesthetizing with MS-222 (Sigma-Aldrich, St. Louis, MO, USA). First, fish were weighed to calculate the weight gain rate (WGR) and specific growth rate (SGR). Color values were then measured by a CR-400 Chroma Meter (Minolta Camera Co. Ltd., Asaka, Japan). Finally, skin samples were dissected from nine fishes in each group and stored at −80 °C for transcriptome and metabolome analysis.

### 2.4. Colorimetric Evaluation

After the feeding trial, each fish was individually photographed with a CR-400 Chroma Meter to analyze the fish body color (Figure S1). The Chroma Meter was used to measure in L*, a*, and b* measurement modes (CIE1976) with the D65 illuminant. The white plate supplied by the manufacturer was used to calibrate the Chroma Meter. The L*a*b* color space (also referred to as CIELAB) is a system for expressing color digitally developed by the International Commission on Illumination (CIE) in 1976. Colors and standardized L*a*b* color space values are matched by this system, where a point in the L*a*b* color space represents the color [42]. In this space, the color parameter L* represents the brightness, and its value ranges from 0 (black) to 100 (white). The a* and b* both represent the color direction. a* > 0 indicates the red direction, a* < 0 indicates the green direction, b* > 0 indicates the yellow direction, and b* < 0 indicates the blue direction. Three repeated measurements were taken on the dorsal skin, ventral skin, dorsal fin, and caudal fin areas of the fish, respectively. All measurements were performed under the same conditions to avoid the effect of daytime on fish body color.

### 2.5. RNA Extraction and Transcriptome Sequencing Analysis

The total RNA of skin was extracted using the TRIzol reagent (Life Technologies, Carlsbad, CA, USA). DNase I (New England Biolabs, Ipswich, MA, USA) was used to remove genomic DNA from RNA samples. RNA quality was assessed using 1% agarose gel electrophoresis and nano spectrophotometer (Nanodrop 2000c, Thermo Scientific, Wilmington, DE, USA). High-quality RNA samples were sent to Biomarker Technologies (Beijing, China) for subsequent library construction and sequencing. Nine cDNA libraries (three pooled samples per group) were constructed from nine RNA samples. Library construction was performed according to the instructions of the NEBNext^®^ Ultra™ RNA Library Prep Kit for Illumina^®^ (NEB, Ipswich, MA, USA). The mRNA was enriched with magnetic beads with Oligo (dT). The mRNA was fragmented by adding fragmentation buffer and the first strand cDNA was synthesized using random hexamer primer. The second strand cDNA was then synthesized using DNA Polymerase I and RNase H. The poly-A base was added to the 3’ end of the cDNA fragment, followed by fragment size selection (240 bp range). Finally, the cDNA library was obtained by PCR enrichment. Library quality was subsequently assessed using the Qsep-400 method on the Agilent Bioanalyzer 2100 system (Agilent Technologies, Palo Alto, CA, USA). The clustering of the index-coded samples was performed on a cBot Cluster Generation System using TruSeq PE Cluster Kit v4-cBot-HS (Illumina, Inc., San Diego, CA, USA) according to the manufacturer’s instructions. The nine constructed libraries were then sequenced on the Illumina NovaSeq 6000 (Illumina, Inc., San Diego, CA, USA) and paired-end reads were generated. After preprocessing the raw data to obtain clean data, the clean data were aligned to the *P. leopardus* reference genome (GCF_008729295.1) using Hisat2 [43]. Fragments per million reads per kilobase (FPKM) were used to calculate gene expression levels. Differential expression analysis was performed for the three comparison groups using the DESeq2R package [44]. The false discovery rate (FDR) and log_2_ fold change (log_2_FC) of genes in the three comparisons were calculated; genes with |log_2_FC| ≥ 1 and FDR < 0.05 were determined as differentially expressed genes (DEGs). The DEGs were mapped to the Gene Ontology (GO) databases and Kyoto Encyclopedia of Genes and Genomes (KEGG, http://www.genome.jp/kegg/, accessed on 7 April 2022) for functional annotation (padj < 0.05).

### 2.6. Metabolome Analysis

After 14 weeks of feeding, nine skin samples were collected from each group, respectively. Skin samples were ground in liquid nitrogen, weighed 60 mg, and mixed with 400 μL of methanol/acetonitrile (1,1, *v*/*v*). The tubes were vortexed for 60 s, underwent 10 min cryogenic ultrasonic extractions, placed at −20 °C for one hour, and centrifuged at 12,000 rpm for 15 min at 4 °C. The supernatant was collected, freeze-dried, and stored at −80 °C for subsequent analysis.

Each sample was analyzed by high performance liquid chromatography using Acquity I-Class PLUS (Waters, Milford, MA, USA) on an Acquity UPLC HSS T3 column (1.8 μm 2.1 × 100 mm, Waters, USA) with an injection volume of 1ul and a flow rate set at 400 μL/min. Both positive and negative ion mobile phases were 0.1% formic acid aqueous solution (A)—0.1% formic acid acetonitrile (B). The Xevo G2-XS QTOF high-resolution mass spectrometer (Waters, USA) was then used for mass spectrometry. Electrospray ion source (ESI) contained two modes of positive and negative ions, where the positive and negative ion capillary voltages were 2500 V and −2000 V, respectively, and the ion source temperature was set to 100 °C and the desolvation gas temperature was set to 500 °C. The mass-to-nucleus ratio (*m*/*z*) acquisition range was 50–1200. Then, principal component analysis (PCA) and orthogonal projections to latent structures discriminant analysis (OPLS-DA) were subsequently performed using multivariate statistical analysis methods to discriminate the accuracy and validity of the data. Metabolites with variable importance in projection (VIP) >1.0 and *p* < 0.05 were determined as significantly different metabolites. The quantitative identification of metabolites was performed with the help of the Kyoto Encyclopedia of Genes and Genomes (KEGG) database, Human Metabolome Database (HMDB) database, and Lipid Metabolites and Pathways Strategy (LIPID MAPS) database were used for annotation.

### 2.7. Quantitative Real-Time PCR (qPCR)

A total of 12 DEGs were randomly selected for real-time PCR evaluation to verify the accuracy of RNA sequencing. RNA samples for RNA-seq library construction were reverse-transcribed using TransScript Uni All-in-One First-Strand cDNA Synthesis SuperMix for qPCR (TransGen Biotech, Beijing, China). The three total RNAs of each group were uniformly configured into 1000 ng for reverse-transcription, and the final reaction volume was 20 µL. Primers designed by Primer Premier software v5.0 (Premier Biosoft, Brandon, MB, Canada) are listed in Supplementary Table S1. Primers were provided by Sangon Biotech Co., Ltd., Shanghai, China. The high-affinity purification (HAP) method was used for the purification of the primers where the primer concentration was 1 OD (5.46 nmol/mL). The qPCR reaction system (total volume 10 µL) consists of 1 µL of template, 0.4 µL each of forward and reverse primers, 3.2 µL of water, and 5 µL of SYBR Green Mix. qPCR was performed on a Light Cycler real-time quantitative PCR system (Roche, Basel, Switzerland) using the PerfectStart Green qPCR SuperMix (TransGen Biotech, Beijing, China). The thermal cycling was performed at 95 °C for 30 s, followed by 40 cycles at 95 °C for 5 s, 56 °C for 20 s, and 72 °C for 20 s. Melting curves were used to analyze the PCR-amplified products of the 12 primers to determine the specificity of the primers. Furthermore, amplification efficiencies and correlation coefficient (R^2^) of each primer were determined by standard curves for 4-fold dilutions (1/4, 1/16,1/64, 1/256, and 1/1024) of the cDNA template (50 ng). Stability analyses of candidate reference genes were also performed by selecting cDNAs from skin of the three groups C, AS, and HP. Afterwards, the average expression stability (M) and coefficient of variation (CV) of the candidate reference genes were analyzed by software such as GeNorm and BestKeeper to select the most stable genes. GeNorm is used to calculate the M value of a gene, where a smaller M value indicates that the gene is more stable. BestKeeper was used to assess the coefficient of variance and standard deviation (SD) of candidate reference genes, where genes with SD < 1 were considered to be stable. Normalization was performed by the delta-delta-CT quantification method [45]. The expression for all the samples were determined in technical triplicates.

### 2.8. Data Calculation and Statistical Analysis

The growth performance parameters of adult *P. leopardus* such as weight gain rate (WGR) and specific growth rate (SGR) were calculated as follows:

WGR (%) = (*W*_t_ – *W*_0_)/*W*_0_ × 100

SGR (%/day) = (ln (*W*_t_/*W*_0_))/*t* × 100

where *W*_t_: mean value of final fish body weight (g), *W*_0_: mean value of initial fish body weight (g), *t*: number of feeding days (days).

All data are expressed as mean ± standard deviation (SD). Experimental data were subjected to one-way ANOVA using IBM SPSS Statistics 24.0 (SPSS Inc., Chicago, IL, USA). Differences between group means were determined by Duncan’s multiple range tests. *p* < 0.05 was considered statistically significant.

## 3. Results

### 3.1. Growth

There was no significant difference (*p* > 0.05) in SGR and WGR between three groups at week 7 (Table 2). SGR and WGR of *P. leopardus* in the HP group were significantly higher than those in the C and AS groups at week 14 (*p* < 0.05), whereas SGR and WGR in the AS group were not significantly increased compared with the C group (*p* > 0.05).

### 3.2. Colorimetric Evaluation

**Figure 1:**
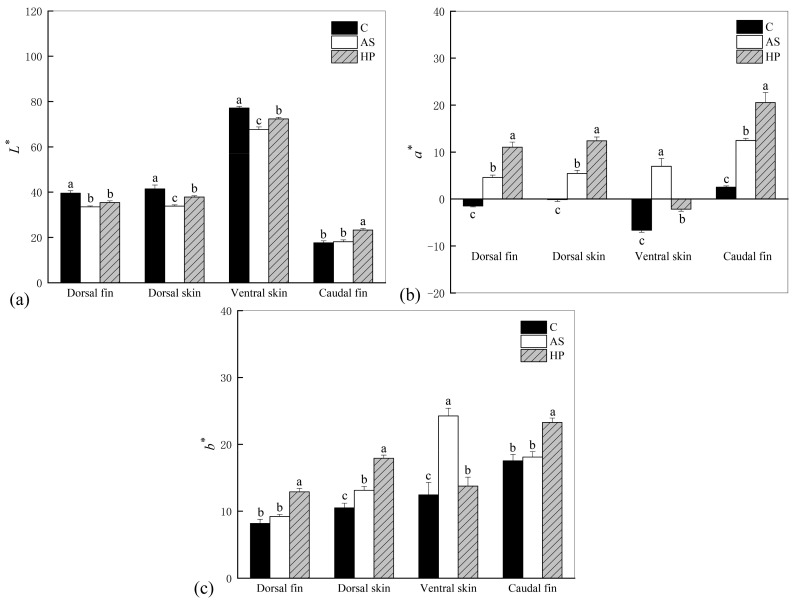
Color parameters of *P. leopardus* after 14 weeks of feeding experimental diets supplemented with different sources of astaxanthin. (**a**) L* indicates brightness—the larger the value, the brighter the body color; (**b**) a* indicates red coloration—positive values represent reddish and negative values represent greenish; (**c**) b* indicates yellow coloration—positive values represent yellowish and negative values represent blueish. Data are presented as mean ± SD. Different letters indicate difference (*p* < 0.05) among groups. The letter C is control, AS is synthetic astaxanthin, HP is natural astaxanthin.

The color parameters L*, a*, and b* of the *P. leopardus* fed for 14 weeks are shown in Figure 1. The brightness (L*) was significantly higher in C than AS and HP in dorsal fin, dorsal skin, and ventral skin (*p* < 0.05). Redness (a*) and yellowness (b*) were significantly higher in HP than C and AS in dorsal fin, dorsal skin, and caudal fin (*p* < 0.05). Among them, both the a* and b* values of the above-mentioned areas were HP > AS > C. However, the values of a* and b* in AS were significantly higher than C and HP in ventral skin (*p* < 0.05).

### 3.3. Transcriptome Analysis

**Figure 2:**
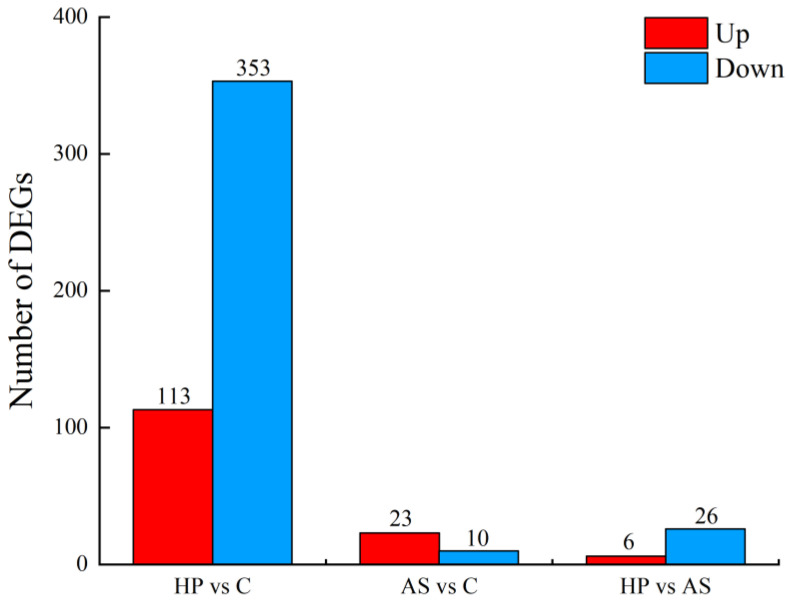
The number of differentially expressed genes in the three comparison groups. Red is the up-regulated genes, blue is the down-regulated genes. The letter C is control, AS is synthetic astaxanthin, HP is natural astaxanthin.

Nine cDNA libraries were constructed (see Table S2 for detailed information). After filtering out low-quality reads and aptamers, 17.82, 17.89, and 17.78 Gb of clean data were obtained for C, AS, and HP, respectively, with an average of 5.94 Gb per sample. The Q30 for each sample was above 93%. The average mapping rate was 92.68%. Compared with expression levels in C, 466 genes were differentially expressed in HP, of which 113 and 353 were up- and down-regulated in the skin of treated fish (Tables S3–S5). A total of 33 DEGs were identified between C and AS, of which 23 and 10 were up- and down-regulated in the skin of treated fish. A total of 32 DEGs were identified between AS and HP, of which 6 and 32 were up- and down-regulated (Figure 2). Candidate genes involved in body color were identified (Table 3). Several genes that may be involved in body color formation in fish were overexpressed in HP compared to C, such as *cyclic-AMP-responsive element-binding protein 3-like 1* (*creb3l1*) and *ATP-binding cassette sub-family C member 6a* (*abcc6a*). In contrast, *fatty-acid-binding protein*, *intestinal* (*fabp2*), and *retinol dehydrogenase 12* (*rdh12*), also associated with pigmentation, showed significant down-regulation in HP. The overexpression of genes involved in melanin synthesis, such as *tyrosinase-related protein 1b* (*tyrp1b*) and *premelanosome protein a* (*pmela*), was observed in AS compared to C. Moreover, *patatin-like phospholipase domain containing 2* (*pnpla2*), *pmela*, *pmelb*, *tyrp1,* and *tyrp1b* showed significant down-regulation in HP compared to AS.

We evaluated DEGs in the three pairwise comparisons between C, AS, and HP by GO and KEGG functional enrichment analyses. In HP vs. C, DEGs were significantly enriched for terms in the biological process category, including muscle contraction (GO:0006936), muscle system process (GO:0003012), muscle cell development (GO:0055001), and sarcomere organization (GO:0045214) (Figure S2). In the biological process category, DEGs in AS vs. C were enriched in circadian regulation of gene expression (GO:0032922). Transcription-factor-activity-related terms were the most common in the molecular function (MF) category for DEGs in AS vs. C (padj < 0.05). Significant GO enrichment was not detected for HP vs. AS owing to the low number of DEGs (padj > 0.05).

**Figure 3:**
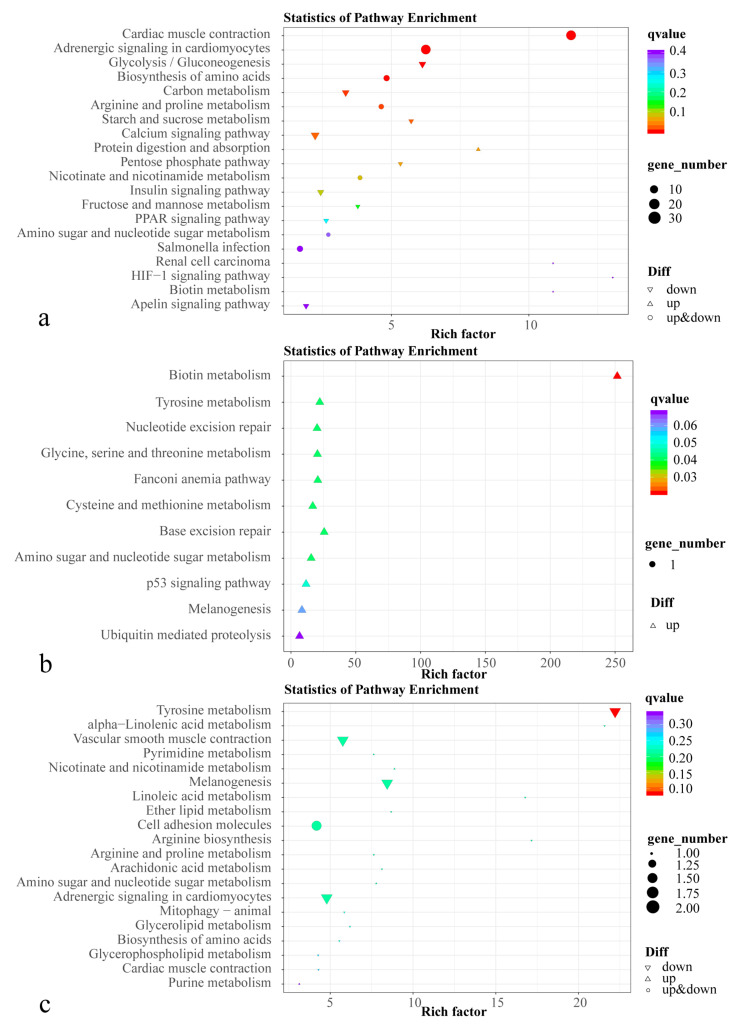
KEGG enrichment of differentially expressed genes in comparisons between C, AS, and HP groups. (**a**) HP vs. C; (**b**) AS vs. C; (**c**) HP vs. AS. The letter C is control, AS is synthetic astaxanthin, HP is natural astaxanthin.

A KEGG enrichment analysis revealed that DEGs in HP vs. C were significantly enriched for 14 pathways, including cardiac muscle contraction, adrenergic signaling in cardiomyocytes, and glycolysis/gluconeogenesis (Figure 3). Six pathways were significantly enriched in AS vs. C, including biotin metabolism, base excision repair, and tyrosine metabolism. Four pathways were significantly enriched in HP vs. AS, including tyrosine metabolism, melanogenesis, α-linolenic acid metabolism, and vascular smooth muscle contraction (*p* < 0.05).

### 3.4. Metabolome Analysis

Based on principal component analysis, there was some overlap between differential metabolites in the HP, AS, and C groups in positive and negative ion modes (Figures S3–S5). The sample categories were further differentiated using OPLS-DA. C and HP, C and AS, and AS and HP showed some separation in both positive and negative ion modes, indicating the stability of LC-MS results (Figure S6).

Compared to levels in the C group, in the positive ion mode, 199 and 157 metabolites were significantly altered in the HP and AS groups, respectively (Table 4). Of these, 188 metabolites increased and 11 metabolites decreased in the HP group. Furthermore, 142 metabolites were increased and 15 metabolites were decreased in the AS group. A total of 105 metabolites were detected between the HP group and AS group, including 75 metabolites increased and 30 metabolites decreased in the HP group. In negative ion mode, compared to levels in the C group, 178 and 92 metabolites were significantly altered in HP and AS, respectively. Among these, 170 metabolites increased and 8 metabolites decreased in the HP group. Additionally, 84 metabolites increased and 8 metabolites decreased in the AS group. In the negative ion mode, compared with levels in AS, 74 metabolites were detected in the HP group, including 59 metabolites increased and 14 metabolites decreased (Tables S6–S8). In the positive ion mode, we did not detect shared differential metabolites in C, AS, and HP (Table 5). In the negative ion mode, four annotated metabolites, 16-hydroxyhexadecanoic acid, 2-hydroxybutyric acid, sepiapterin, and uridine, were differentially changed in all comparisons.

A KEGG enrichment analysis of differential metabolites was performed. Compared with C, the main metabolic pathways enriched in HP were α-linolenic acid metabolism and vitamin digestion and absorption (Table 6). AS was mainly enriched by pentose and glucuronate interconversions, glutathione metabolism, ferroptosis, and thyroid hormone synthesis. In HP vs. AS, the key metabolic pathways were nonribosomal peptide structures and 2-oxocarboxylic acid metabolism.

### 3.5. Integrated Transcriptome and Metabolome Analysis

Combined metabolomic and transcriptomic analyses showed that differential genes and metabolites in the HP group were enriched in lipid-metabolism-related pathways, including α-linolenic acid metabolism, arachidonic acid metabolism, linoleic acid metabolism, glycerophospholipid metabolism, and other lipid metabolism (Figure S7). Nucleotide metabolism, including purine metabolism and pyrimidine metabolism, was also enriched. Notably, the HP group was also enriched for glycerophospholipid metabolism, ether lipid metabolism, arachidonic acid metabolism, and other pathways related to lipid metabolism as well as pyrimidine metabolism, compared to the AS group. In the C and AS comparison, only biotin metabolism, cysteine and methionine metabolism, and amino sugar and nucleotide sugar metabolism were enriched.

### 3.6. Validation of Gene Expression Levels by qPCR

**Figure 4:**
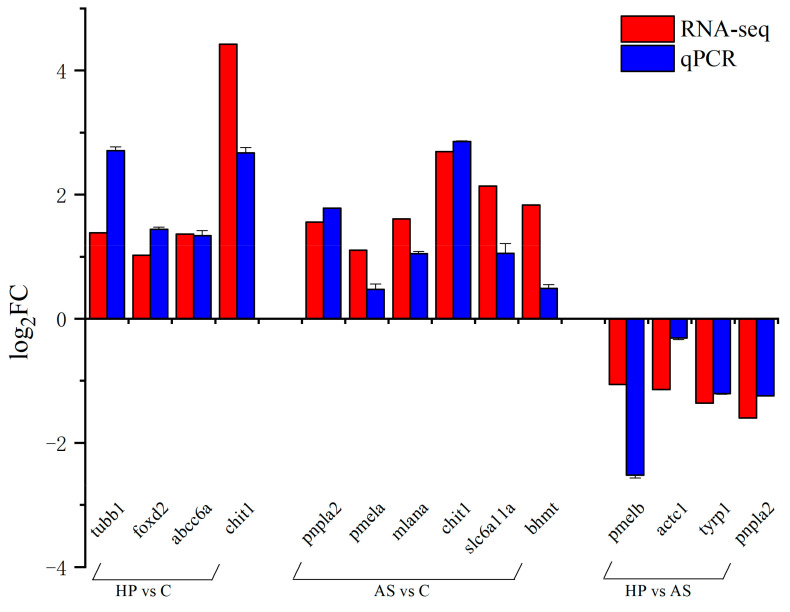
Comparison of the expression levels of DEGs by qPCR and RNA-seq. The x-axis is the gene name, and the y-axis indicates the log_2_FC of genes. Data are presented as mean ± SD. The letter C is control, AS is synthetic astaxanthin, HP is natural astaxanthin, qPCR is quantitative real-time PCR.

The analysis of melting curves showed that all primers showed a single peak, and the Tm corresponding to their peaks were all higher than 80 °C, which indicated that the primers had better specificity (Table S12). Additionally, amplification efficiency of all primers ranged from 90–105% and the correlation coefficient of the standard curve was ≥0.993, indicating that the primers met the requirements of qPCR and could be used for subsequent experiments (Table S1). The results of GeNorm showed that the gene *eukaryotic elongation factor 2* (*eef2*) had the lowest M value (Table S12). Additionally, the gene *eef2* had a small CV value in the results of the BestKeeper analysis. The SD of the gene *eef2* was also the smallest and the SD < 1 (Table S12). These results demonstrate the better stability of the gene *eef2*. The gene *eef2* was used as a reference gene (M = 0.58, CV = 4.49%). Relative expression analysis was performed on 12 genes and the expression trends for these genes were similar to the results in the RNA-seq profiles (Figure 4). This demonstrates the reliability of the RNA-seq data.

## 4. Discussion

The role of carotenoids in promoting growth in fish is controversial. Zhu et al. [26] found no significant increase in the growth performance of *P. leopardus* after treatment with different concentrations of astaxanthin. Micah et al. [46] found that astaxanthin had no significant effect on blood parrotfish growth. At week 14, WGR and SGR of fish in the HP group were significantly higher than those in the C and AS groups. This suggests that natural astaxanthin exerted a beneficial effect on the growth of *P. leopardus*, which is similar to results for other species, such as *Salmo salar* [47], *Pagrus pagrus* [48], and *Pseudosciaena crocea* [49]. DEGs between the HP and C group were involved in energy-metabolism-related pathways, such as sulfur metabolism and oxidative phosphorylation. In particular, *cytochrome c oxidase subunit 7A2* (*cox7a2*), *NADH:ubiquinone oxidoreductase subunit B3* (*ndufb3*), and *selenium-binding protein 1* (*selenbp1*) were down-regulated; lipid metabolism-related pathways were also enriched. These results suggest that *P. leopardus* may have a higher absorption and utilization efficiency for natural astaxanthin. Previous studies have found that astaxanthin can enhance the intermediate metabolism to promote the absorption and utilization of nutrients, thus improving the growth performance of rainbow trout [50]. However, in our study, synthetic astaxanthin did not have a significant effect on the growth performance of *P. leopardus*. Additionally, natural astaxanthin did not show a growth-promoting effect on the species in the seventh week. The growth of fish may also be related to the development stage, breeding environment, and breeding time. It is important to note that other ingredients in the supplement may also provide a supporting role in growth, even though we have maintained the consistency of the astaxanthin, protein, and fat content, respectively. Hence, the mechanism by which astaxanthin may affect the growth performance of fish needs to be further investigated.

We found that astaxanthin decreased skin brightness L*. The addition of astaxanthin to the diet effectively increased the redness a* and yellowness b* in *P. leopardus*. The decrease in fish brightness may be related to astaxanthin. Some studies have shown that astaxanthin supplementation increased redness and yellowness but decreased brightness in blood parrotfish and *Colisa lalia* [46,51]. This suggests that astaxanthin deposition may promote fish coloration but also lead to a reduction in brightness, resulting in a dark red appearance of the fish. Moreover, the beneficial effect of natural astaxanthin on *P. leopardus* color was significantly greater than that of synthetic astaxanthin, and a* and b* values in the HP fish were significantly higher than those in the AS group. This may be related to the efficiency of astaxanthin digestion as well as absorption and tissue deposition. Tissue deposition in *P. leopardus* may be more effective for natural astaxanthin. We also found that the body color of fish fed synthetic astaxanthin was weaker than that of the fish fed natural astaxanthin. In addition to stereochemistry, natural and synthetic astaxanthin also differ in esterification. Natural astaxanthin is mainly present in the esterified form, with a small percentage in the free state. Synthetic astaxanthin, however, is present in the non-esterified free form [25]. Angell et al. [52] found that the esterified astaxanthin was better absorbed than the free pigment, resulting in better coloration compared to that of the free form of synthetic astaxanthin. In *Exopalaemon carinicauda*, natural astaxanthin from *H. lacustris* showed better efficiency in terms of pigmentation [53], consistent with the results of the present study. Furthermore, Maoka et al. found higher levels of total carotenoids in the red skin of *P. leopardus* than in brown and black fish. The red skin of this species contained 52.7% and 28.5% of the total carotenoids in astaxanthin diesters and astaxanthin monoesters, respectively, while only 0.8% of astaxanthin diesters were detected in the black skin [54]. This suggests that red color development *P. leopardus* skin cannot be achieved without esterified astaxanthin. Li et al. found a consistency between the intensity of skin attaining a red color in blood parrots and the concentration of astaxanthin in the diet. The rate of fish attaining a red color accelerated with increasing astaxanthin in the diet. Additionally, the coloring effect of synthetic astaxanthin was better than that of natural astaxanthin [55]. However, there was no consistency in the amount of astaxanthin added in their study, which resulted in diets with much higher levels of synthetic astaxanthin than natural astaxanthin. As they mentioned, a similar coloring effect can be obtained with natural astaxanthin if the content of natural astaxanthin is consistent with that of synthetic astaxanthin. In the experiments, more *H. lacustris* powder was added to the HP diet in order to obtain a uniform astaxanthin content. However, it is unclear whether other ingredients in the supplement also contribute to fish coloration, so further research is needed.

Red, orange, and yellow tissue colors in most animals are determined by carotenoids and pteridine pigments. Unlike melanin and pteridine pigments, carotenoids cannot be synthesized by fish de novo and need to be obtained in the diet [56,57]. As a fat-soluble substance, the carotenoid transport process is closely associated with the transport of other lipids. Binding to specific lipoproteins or apolipoproteins is necessary to transport carotenoids to specific tissues for deposition [58,59]. In our study, the PPAR signaling pathway, related to lipid metabolism, was significantly enriched; various genes in this pathway, including *acyl-CoA synthetase long-chain family member 1a* (*acsl1a*), *carnitine palmitoyl transferase 1b* (*cpt1b*), *fabp2,* and *malic enzyme 1* (*me1*), were significantly down-regulated. ACSLs (long-chain fatty acyl-CoA synthetases) are essential enzymes for the catalytic production of esteryl coenzyme A from fatty acid activation in mammals. It plays an important role in the synthesis of cholesterol, triglycerides, and phospholipids, and in fatty acid metabolism [60]. The overexpression of this gene promotes the transport of fatty acids and the deposition of triglycerides [61,62,63]. Gene *cpt1b* reduces fat deposition by regulating the entry of long-chain fatty acids into mitochondria for β-oxidation [64]. Fatty-acid-binding proteins play a key role in fatty acid uptake, intracellular transport, and metabolism [65]. Chicken *fabp2* expression promotes fat deposition [66]. Samulin et al. [67] found that the co-expression of *fabp2* and *fabp1* may also have important effects on the regulation of fat deposition in pigs. me1 provides reductase for fatty acid biosynthesis coenzyme II (NADPH), which makes a positive contribution to lipid synthesis [68]. In the present study, *acsl1a*, *cpt1b*, *fabp2*, and *me1* were significantly down-regulated in HP vs. C, which may lead to the inhibition of fatty acid catabolic activity in fish in the HP group. Furthermore, we found that the expression of *pnpla2*, which is associated with lipid storage and metabolism, was down-regulated in HP vs. AS. The gene *pnpla2* encodes adipose triglyceride lipase (ATGL), which catalyzes the hydrolysis of triglyceride to diacylglycerol and free fatty acids. Studies of East African cichlids and mutant rainbow trout have reported that *pnpla2* expression is up-regulated in yellow skin. This suggests that *pnpla2* may facilitate carotenoid storage and promote yellow pigment deposition [17,69]. Additionally, the gene *rdh12,* related to retinol metabolism using carotenoids as substrates, was down-regulated in HP vs. C, which may explain the low carotenoid substrate conversion in the fish skin.

Arachidonic acid in feed is also associated with pigmentation in fish. Diets with excessive arachidonic acid affect pigmentation in fish and can lead to albinism [70]. In the arachidonic acid metabolic pathway of HP vs. C, we found that *Cytochrome P450 2K1* (*cyp2k1*) expression was down-regulated while *phospholipase A2 group X* (*pla2g10*) expression was up-regulated. *Phospholipase A2 group III* (*pla2g3*) gene expression was down-regulated in HP vs. AS. The cytochrome CYP2 family is involved in the oxidation of fatty acids, such as arachidonic acid and lauric acid [71]. The gene *cyp2k1* has significant fatty acid hydroxylase activity [72]. *cyp2k1* gene down-regulation in HP might inhibit fatty acid oxidation. The gene *pla2g10* catalyzes the hydrolysis of glycerophospholipids and the production of arachidonic acid [73]. The up-regulation of *pla2g10* in the HP group may correspond to an increase in the arachidonic acid content. The gene *pla2g3* is involved in the hydrolysis of arachidonic acid on the sperm membrane in mice [74]. We found that *pla2g3* expression was down-regulated in the HP group compared to AS, suggesting that diets supplemented with natural astaxanthin may cause the inhibition of arachidonic acid hydrolysis. Arachidonic acid is converted to prostaglandin- and thromboxane-like metabolites via cyclooxygenase. The final conversion in the liver generates the metabolite 11-dehydro-thromboxane B2. Moreover, 5(S)-HETE and leukotriene are lipoxygenase metabolites resulting from arachidonic acid metabolism [74,75]. Increased levels of metabolites 11-dehydro-thromboxane B2 and 5(S)-HETE implies that arachidonic acid depletion is enhanced in the HP group. We speculate that arachidonic acid may be required for color enhancement in *P. leopardus*. The depletion of arachidonic acid thereby contributes to increased skin redness, similar to the results of Zhu et al. [76].

In the present study, we found that KEGG signaling pathways associated with melanin synthesis, such as tyrosine metabolism and melanogenesis, were significantly enriched in HP compared to AS, of which *tyrp1* and *tyrp1b* were significantly down-regulated. Tyrosine metabolism is associated with the synthesis of melanin, especially eumelanin. The tyrosinase protein family, mainly composed of *tyr*, *tyrp1,* and *tyrp2*, includes key enzymes involved in the regulation of melanin synthesis. It plays an important role in melanogenesis in the skin and hair of animals. The production of albino phenotypes in vertebrates is caused by mutations or deletions in the *tyrp1* gene [77]. Lin et al. found that the expression level of *tyrp1* was higher in black-spotted skin than in non-spotted skin in *Scatophagus argus* [78]. The gene *pmel*/*silv* is located downstream of the tyrosine gene family in the melanin synthesis pathway, which plays an important role in the formation of melanosomes [79]. The knockdown of the *pmela* or *pmelb* gene in Nile tilapia results in a significant reduction in the number and size of melanocytes in the skin [80]. The expression level of *creb3* was more highly expressed in HP than in C. Studies have shown that the activation of creb induces the expression of *mitf*, a downstream gene. The expression of mitf can also promote the expression of the downstream genes *tyr*, *tyrp1,* and *tyrp2* and finally promote the generation of melanin [81]. In the present study, the expression levels of *tyrp1* and *pmel* were significantly lower in HP than in AS, suggesting that melanogenesis may be suppressed, to some extent, and that the skin was significantly redder in HP. The *tyrp1* and *pmel* levels were higher in AS than in C. This suggests that the addition of synthetic astaxanthin contributed to the production of melanin in the fish. However, the redness a* was also significantly higher in AS than in C. Therefore, further studies are needed to determine whether the inhibition of melanin synthesis or the reduction in the melanin content in the skin contributed to the redder phenotype of *P. leopardus*.

In the present study, we found that the ABC transporter pathway was enriched in all three comparisons, suggesting that ABC transporters play an important role in the uptake and transport of carotenoids. Increased levels of ABC-transporter-protein-associated inosine and uridine in the HP group, compared to the control group. Inosine is involved in ATP production as a raw material for energy synthesis. It is degraded by glial cells to uric acid, which acts as a physiological antioxidant to reduce oxidative damage [82,83]. Similar to the metabolomic data, a transcriptome analysis revealed the significant up-regulation of *abcc6a* expression in the HP group. We hypothesized that ABC transporters are involved in pigment substrates that contribute to red pigment deposition in fish. We also detected significant enrichment for glutathione metabolism and thyroid hormone synthesis pathways in both AS and HP, with the increased levels of the associated metabolite oxidized glutathione. As an endogenous antioxidant, glutathione is able to scavenge excess reactive oxygen species produced during oxidative stress. Glutathione inhibits tyrosinase activity and slows down the oxidation of L-tyrosine to form melanin, thus reducing the melanin content in the organism [84]. This is consistent with the down-regulation of melanin-synthesis-related genes (*pmel* and *tyrp1*) in the HP group.

In the present study, levels of *γ-glutamyl hydrolase* (*ggh*) and the metabolites sepiapterin and 4a-hydroxytetrahydrobiopterin in the folate biosynthesis pathway were higher in HP than in C. The gene *ggh* converts 5 methyl and 10-formyl-tetrahydrofolate forms of folic acid to the monoglutamic form in the intestine, allowing its transport through biofilms [85]. It is noteworthy that the differentially expressed genes and metabolites in all three comparisons were enriched for folate biosynthesis pathway. The content of the metabolite sepiapterin in this pathway is increased. Sepiapterin is a yellow pteridine-like pigment involved in insect body color formation. Studies of zebrafish embryonic pteridine pigments have revealed that sepiapterin is catalyzed by sepiapterin reductase (SPR) to produce tetrahydrobiopterin (BH_4_) [86]. BH_4_ can be oxidized to 4a-hydroxytetrahydrobiopterin, which can also contribute to 4a-carbinolamine dehydratase and dihydropteridine reductase reduction under the action of BH_4_ [87,88]. This suggests that sepiapterin and 4a-hydroxytetrahydrobiopterin may be involved in the formation and deposition of red and yellow pigments in *P. leopardus*. However, no DEGs related to the pteridine pathway were found in the transcriptome analysis. Therefore, the functions of these metabolites in fish still need to be further investigated.

## 5. Conclusions

We conducted a comparative analysis of the coloration of *P. leopardus* fed natural and synthetic astaxanthin and found that astaxanthin had a beneficial effect on fish coloration. Natural astaxanthin resulted in greater increases in the red and yellow values of the fish skin than those of synthetic astaxanthin. Transcriptome and metabolome analyses showed that the melanin synthesis pathway contributes to coloration in *P. leopardus*. Natural astaxanthin inhibited the synthesis of melanin, while synthetic astaxanthin may promoted the synthesis of melanin, which may explain why fish in the natural astaxanthin group had darker coloration than that of fish in the synthetic astaxanthin group. In addition, we also detected significant enrichment for pathways related to carotenoid uptake and transport as well as lipid metabolism. These results support the important role of carotenoids in fish coloration and provide a theoretical basis for color enhancement during the industrial culture of *P. leopardus*.

## Figures and Tables

**Table 1:** Ingredients and proximate composition of the experimental diets.

	Diets		
Ingredients (%)	C	AS	HP
Fish meal	60	60	60
Wheat gluten flour	5	5	5
Soybean meal	5	5	5
Seaweed powder	3	3	3
Wheat meal	14.5	14.5	14.5
Soybean lecithin	1	1	1
Fish oil	4	4	4
Ca(H_2_PO_4_)_2_	1.5	1.5	1.5
Vitamin premix and mineral premix	1	1	1
Microcrystalline cellulose	5	4.8	4.333
Carophyll^®^ pink powder	0	0.2	0
*Haematococcus lacustris* powder	0	0	0.667
Total	100	100	100
Proximate composition (%, dry matter)			
Moisture	7.3	7.8	7.4
Crude protein	53.4	52.7	52.2
Lipids	10.8	10.7	11.2
Ash	12.2	13.4	13.3
Astaxanthin	0	0.0193	0.0186

Note: 1. Fish meal, wheat gluten flour, soybean meal, seaweed powder, wheat meal, soybean lecithin, fish oil, Ca(H2PO_4_)_2_ were purchased from Guangdong Yuehai Biotechnology Co., Ltd., Zhanjiang, China. 2. Astaxanthin content in Carophyll^®^ pink powder accounted for 10%. Carophyll^®^ pink powder was produced by Guangzhou Leader Biotechnology Co., Ltd., Guangzhou, China. 3. Astaxanthin content in *Haematococcus lacustris* powder accounted for 3%. The *H. lacustris* powder was produced by Yunnan Alphy Biotechnology Co., Ltd., Chuxiong, China. 4. The letter C is control, AS is synthetic astaxanthin, HP is natural astaxanthin.

**Table 2:** The growth performance of *P. leopardus* during the 14-week feeding experiment.

	Treatment	WGR (%)	SGR (%/Day)
7 weeks	C	33.91 ± 3.94 ^a^	0.59 ± 0.06 ^a^
	AS	33.71 ± 15.70 ^a^	0.55 ± 0.24 ^a^
	HP	39.82 ± 8.00 ^a^	0.67 ± 0.11 ^a^
14 weeks	C	66.07 ± 3.24 ^a^	0.52 ± 0.02 ^a^
	AS	76.04 ± 9.92 ^a^	0.57 ± 0.06 ^a^
	HP	98.94 ± 8.05 ^b^	0.70 ± 0.04 ^b^

Note: Data are presented as mean ± SD (*n* = 9). Columns with different letters indicate difference (*p* < 0.05) among groups. The letter C is control, AS is synthetic astaxanthin, HP is natural astaxanthin, WGR is weight gain rate, and SGR is specific growth rate.

**Table 3:** Differentially expressed genes associated with body color.

Comparison	Gene ID	Gene Name	Gene Annotation	Log_2_FC	Regulated
HP vs. C	gene-LOC121962187	*fabp2*	fatty-acid-binding protein, intestinal-like	−2.50	down
	gene-fabp2	*fabp2*	fatty-acid-binding protein, intestinal	−1.85	down
	gene-*creb3*l1	*creb3l1*	cyclic AMP-responsive element-binding protein 3-like protein 1	1.09	up
	gene-abcc6a	*abcc6a*	multidrug-resistance-associated protein 1	1.36	up
	gene-*rdh12*	*rdh12*	retinol dehydrogenase 12-like	−1.65	down
AS vs. C	gene-*tyrp1b*	*tyrp1b*	5,6-dihydroxyindole-2-carboxylic acid oxidase-like	1.91	up
	gene-pmela	*pmela*	melanocyte protein PMEL-like	1.35	up
HP vs. AS	gene-LOC121950639	*pnpla2*	patatin-like phospholipase-domain-containing protein 2	−1.60	down
	gene-pmela	*pmela*	melanocyte protein PMEL-like	−1.21	down
	gene-LOC121963361	*pmelb*	premelanosome protein b	−1.06	down
	gene-LOC121962312	*tyrp1*	5,6-dihydroxyindole-2-carboxylic acid oxidase	−1.36	down
	gene-*tyrp1b*	*tyrp1b*	5,6-dihydroxyindole-2-carboxylic acid oxidase-like	−1.58	down

Note: The letter C is control, AS is synthetic astaxanthin, HP is natural astaxanthin. Gene ID information is available in Supplementary Tables S3–S5.

**Table 4:** Number of significantly different metabolites among different groups.

Group	Ion Mode	Up	Down	All
HP vs. C	POS	188	11	199
AS vs. C	POS	142	15	157
HP vs. AS	POS	75	30	105
HP vs. C	NEG	170	8	178
AS vs. C	NEG	84	8	92
HP vs. AS	NEG	59	15	74

Note: The letter C is control, AS is synthetic astaxanthin, HP is natural astaxanthin. POS indicates the positive ion mode; NEG indicates the negative ion mode.

**Table 5:** Common differential metabolites in HP and C, AS and C, HP and AS.

Metabolite ID	Name	Mass Error (ppm)	*m*/*z*	RT (min)
neg_5462	16-Hydroxyhexadecanoic acid	3.66	271.23	7.93
neg_1491	2-Hydroxybutyric acid	15.15	103.04	0.85
neg_3002	Sepiapterin	−0.61	511.14	1.43
neg_2991	Uridine	6.13	243.06	1.42

Note: The letter C is control, AS is synthetic astaxanthin, HP is natural astaxanthin, *m*/*z* is mass to charge ratio, RT is retention time. Metabolite ID information is available in Supplementary Tables S6–S8.

**Table 6:** KEGG pathway enrichment of metabolites with statistically significant abundance changes.

Comparison	ko_ID	KEGG Pathway	Ion Mode	*p*-Value
HP vs. C	ko00592	α-Linolenic acid metabolism	POS	4.24 × 10^−3^
	ko04977	Vitamin digestion and absorption	POS	3.24 × 10^−2^
AS vs. C	ko00040	Pentose and glucuronate interconversions	POS	3.60 × 10^−2^
	ko00480	Glutathione metabolism	NEG	2.04 × 10^−2^
	ko04216	Ferroptosis	NEG	2.04 × 10^−2^
	ko04918	Thyroid hormone synthesis	NEG	2.04 × 10^−2^
HP vs. AS	ko01054	Nonribosomal peptide structures	POS	3.90 × 10^−2^
	ko01210	2-Oxocarboxylic acid metabolism	POS	4.97 × 10^−2^

Note: The letter C is control, AS is synthetic astaxanthin, HP is natural astaxanthin. POS indicates the positive ion mode; NEG indicates the negative ion mode. The ko_ID means KEGG metabolic pathway ID from Supplementary Tables S9–S11.

## Data Availability

The RAD-seq data were deposited in the sequence read archive (SRA) database under accession number PRJNA89959. The data that support the study findings are available upon request.

## Supplementary Materials

The following supporting information can be downloaded at: https://www.mdpi.com/article/10.3390/ani13071252/s1, Figure S1: Measurement areas of body color parameter *L**, *a**, *b** of leopard coralgrouper; Figure S2: GO enrichment analysis of three comparative groups of DEGs. (a) HP vs. C, (b) AS vs. C, (c) HP vs. AS; Figure S3: Principal component analysis (PCA) score plots of HP and C groups in positive (a) and negative (b) ion mode. The circle symbol represents group C and the triangle symbol represents group HP; Figure S4: Principal component analysis (PCA) score plots of AS and C groups in positive (a) and negative (b) ion mode. The triangle symbol represents group C and the circle symbol represents group AS; Figure S5: Principal component analysis (PCA) score plots of HP and AS groups in positive (a) and negative (b) ion mode. The circle symbol represents group AS and the triangle symbol represents group HP; Figure S6: Orthogonal partial least squares discriminant analysis (OPLS-DA) score plots for three comparison groups. (a,b) OPLS-DA score plots of HP and C following positive (a) and negative (b) modes. (c,d) OPLS-DA score plots of AS and C following positive (c) and negative (d) modes. (e,f) OPLS-DA score plots of HP and AS following positive (e) and negative (f) modes; Figure S7: KEGG enrichment pathways in DEGs and DMs in the comparison group. (a) HP vs. C, (b) AS vs. C, (c) HP vs. AS; Table S1: Sequences of DEGs for RT-qPCR relative expression analysis; Table S2: Summary statistics of skin transcriptome sequencing data in *P. leopardus*; Table S3: Differentially expressed genes in the skin between HP and C; Table S4: Differentially expressed genes in the skin between AS and C; Table S5: Differentially expressed genes in the skin between HP and AS; Table S6: Differential metabolites in the skin between HP and C in positive and negative ion modes; Table S7: Differential metabolites in the skin between AS and C in positive and negative ion modes; Table S8: Differential metabolites in the skin between HP and AS in positive and negative ion modes; Table S9: KEGG pathways annotated by differential metabolites between HP and C in positive and negative ion mode; Table S10: KEGG pathways annotated by differential metabolites between AS and C in positive and negative ion mode; Table S11: KEGG pathways annotated by differential metabolites between HP and AS in positive and negative ion mode; Table S12: Analysis results of expression stability of candidate reference genes.
